# Impact on postoperative complications of changes in skeletal muscle mass during neoadjuvant chemotherapy for gastro‐oesophageal cancer

**DOI:** 10.1002/bjs5.50331

**Published:** 2020-08-25

**Authors:** R. B. den Boer, K. I. Jones, S. Ash, G. I. van Boxel, R. S. Gillies, T. O'Donnell, J. P. Ruurda, B. Sgromo, M. A. Silva, N. D. Maynard

**Affiliations:** ^1^ Departments of Surgery University Medical Centre Utrecht, Utrecht University Utrecht the Netherlands; ^2^ Oxford University Hospitals NHS Trust, Oxford University Oxford UK; ^3^ Portsmouth Hospitals NHS Trust Portsmouth UK

## Abstract

**Background:**

Risk assessment is relevant to predict postoperative outcomes in patients with gastro‐oesophageal cancer. This cohort study aimed to assess body composition changes during neoadjuvant chemotherapy and investigate their association with postoperative complications.

**Methods:**

Consecutive patients with gastro‐oesophageal cancer undergoing neoadjuvant chemotherapy and surgery with curative intent between 2016 and 2019 were identified from a specific database and included in the study. CT images before and after neoadjuvant chemotherapy were used to assess the skeletal muscle index, sarcopenia, and subcutaneous and visceral fat index.

**Results:**

In a cohort of 199 patients, the mean skeletal muscle index decreased during neoadjuvant therapy (from 51·187 to 49·19 cm^2^/m^2^; *P* < 0·001) and the rate of sarcopenia increased (from 42·2 to 54·3 per cent; *P* < 0·001). A skeletal muscle index decrease greater than 5 per cent was not associated with an increased risk of total postoperative complications (odds ratio 0·91, 95 per cent c.i. 0·52 to 1·59; *P* = 0·736) or severe complications (odds ratio 0·66, 0·29 to 1·53; *P* = 0·329).

**Conclusion:**

Skeletal muscle index decreased during neoadjuvant therapy but was not associated with postoperative complications.

## Graphical abstract

Deconditioning during neoadjuvant chemotherapy is a possible parameter for predicting operative outcomes. This retrospective cohort study aimed to assess body composition changes in patients with gastro‐oesophageal cancer during neoadjuvant therapy and investigate their association with postoperative complications. Skeletal muscle index values decrease during neoadjuvant therapy, but this change is not associated with postoperative complications.

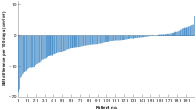

Does not predict risk of complications

## Abstract (es)

**Antecedentes:**

La evaluación de riesgo es importante para predecir los resultados postoperatorios en pacientes con cáncer gastroesofágico. Este estudio de cohortes tuvo como objetivo evaluar los cambios en la composición corporal durante la quimioterapia neoadyuvante e investigar su asociación con complicaciones postoperatorias.

**Métodos:**

Los pacientes consecutivos con cáncer gastroesofágico sometidos a quimioterapia neoadyuvante y cirugía con intención curativa entre 2016 y 2019, identificados a partir de una base de datos específica, se incluyeron en el estudio. Se utilizaron las imágenes de tomografía computarizada, antes y después de la quimioterapia neoadyuvante, para evaluar el índice de masa muscular esquelética, la sarcopenia y el índice de grasa visceral y subcutánea.

**Resultados:**

En una cohorte de 199 pacientes, el índice de masa muscular esquelética disminuyó durante el tratamiento neoadyuvante (de 51,87 cm^2^/m^2^ a 49,19 cm^2^/m^2^, *P* < 0,001) y las tasas de sarcopenia aumentaron (de 42,2% a 54,2%, *P* < 0,001). Una disminución del índice de masa muscular esquelética > 5% no se asoció con un mayor riesgo de complicaciones postoperatorias globales (razón de oportunidades, *odds ratio*: 0,908; ic. del 95%: 0,520‐1,587, *P* = 0,736) ni de complicaciones graves (*odds ratio*: 0,661; i.c. del 95%: 0,286‐1,525, *P* = 0,329).

**Conclusión:**

El índice de masa muscular esquelética disminuyó durante el tratamiento neoadyuvante, pero no se asoció con complicaciones postoperatorias.

## Introduction

Gastro‐oesophageal cancer surgery is still associated with high rates of postoperative morbidity^1^. Historically, cancers affecting the upper gastrointestinal tract are associated with significant malnutrition, which may in part explain the increased surgical morbidity observed^2^. The majority of patients with surgically resectable disease receive neoadjuvant chemotherapy or chemoradiotherapy^3^. It has been suggested that neoadjuvant therapy (NAT) may contribute to alterations in body composition, including a reduction in muscle mass, leading to increased postoperative morbidity^4^, ^5^.

Sarcopenia is the progressive and generalized loss of muscle mass and function. It is most commonly associated with ageing, but cancer cachexia can also contribute^6^. Although dual‐energy X‐ray absorptiometry is the most accurate method for measuring lean muscle mass, alternative methods have been proposed, including CT, which is performed routinely for cancer staging. Recent systematic reviews^7^, ^8^ have demonstrated that preoperative CT‐assessed sarcopenia is predictive for postoperative complications and disease‐specific mortality in patients with oesophageal and gastric cancer. Sarcopenia is potentially modifiable, and may be targeted with prehabilitation strategies. Loss of muscle mass during NAT is associated with adverse clinical outcomes in metastatic colonic and ovarian cancer, but results in gastro‐oesophageal cancer have been inconsistent^9^, ^10^.

The primary aim of this study was to assess changes in body composition during neoadjuvant chemotherapy and to determine its predictive value for postoperative complications in patients with gastro‐oesophageal cancer.

## Methods

The study was approved by the National Research Ethics Committee (Integrated Research Application System number 260643, research ethics committee reference 19/LO/1871).

Consecutive patients with gastro‐oesophageal cancer who had neoadjuvant chemotherapy and surgical resection between March 2016 and June 2019 were identified for eligibility from a specific database. Patients were included if they had a diagnosis of gastric or oesophageal cancer, were over 18 years old and had received neoadjuvant chemotherapy before surgery with curative intent. Patients without eligible CT images for assessment of body composition before and after NAT were excluded.

Clinicopathological data included age, height, weight, ASA grade, co‐morbidity, tumour site, stage and cell type, chemotherapy details (regimen and cycles received) and surgical details (procedure subtype and approach). Complications were collected according to an international consensus statement and scored using the Clavien–Dindo classification method^11^, ^12^. Thirty‐ and 90‐day mortality rates and overall survival data were obtained from the same database.

### Measurement of body composition

CT scans obtained before NAT and before surgery were analysed. A cross‐sectional slice at the level of L3 was extracted and analysed by CoreSlicer (https://coreslicer.com/), a validated web‐based tool to assess body composition semiautomatically. The CoreSlicer tool uses Hounsfield unit ranges of −190 to −30 for fat tissue and − 29 to 150 for skeletal muscle^13^. Body composition was assessed by two independent and blinded investigators. All musculature and adipose tissue was analysed. Final images of the results of body composition assessment were checked for accuracy by another blinded researcher.

The muscle area was divided by the height squared to calculate the skeletal muscle index (SMI) (cm^2^/m^2^)^14^. Similarly, visceral and subcutaneous fat indices were calculated. The SMI limit for sarcopenia was less than 52·4 cm^2^/m^2^ for men and less than 38·5 cm^2^/m^2^ for women, according to cut‐off values used elsewhere^15^. Changes in SMI during NAT were calculated, and patients divided into two groups: SMI decrease during NAT of 5 per cent or less and more than 5 per cent.

### Statistical analysis

All statistical analyses were performed in SPSS® version 25.0 (IBM, Armonk, New York, USA). Continuous variables were tested for normality using the Shapiro–Wilk test. Normally distributed data are presented as mean(s.d.) values, and non‐normally distributed data as median (i.q.r.) values.

To compare paired patient groups the paired *t* test was used for normally distributed data, Wilcoxon test for non‐normally distributed data, and McNemar's test for binomial data. In non‐paired groups an independent Student's *t* test, Mann–Whitney *U* test and the χ^2^ test were used for normally distributed, non‐normally distributed and binomial data respectively. Fisher's exact test was used instead of the χ^2^ test when two or more cells included fewer than five events. The χ^2^ test was used to calculate odds ratios (ORs) with binomial variables in the univariable analysis. Ultimately, logistic regression analysis was used for the multiple regression analyses of postoperative complications. Variables with clinical relevance were deemed eligible for inclusion in the multiple regression analysis.

## Results

Characteristics of the included patients are presented in *Table* *1*. After exclusion of one patient without eligible CT images, a total of 199 patients were included. The majority were men (79·4 per cent) and most tumours were located at the gastro‐oesophageal junction (53·3 per cent). The remaining patients had either oesophageal (34·2 per cent) or gastric (12·6 per cent) tumours. Some 177 patients (88·9 per cent) had an open surgical procedure and 22 (11·1 per cent) of the operations were laparoscopic or thoracoscopically assisted.

**Table 1:** Patient characteristics

		Decrease in SMI (%)		
	Total (*n* = 199)	**≤ 5 (*n* = 108)**	**> 5 (*n* = 91)**	*P* ‡
**Age (years)** *	66·1 (28·4–80·0)	65·6 (45·3–80·0)	67·6 (33·5–79·5)	0·009
**No. of men**	158 (79·4)	77 (71·3)	81 (89)	0·002
**ASA grade**				0·614
I	16 (8·0)	10 (9·3)	6 (7)	
II	128 (64·3)	69 (63·9)	59 (65)	
III	55 (27·6)	29 (26·9)	26 (29)	
**Co‐morbidity**				
None	54 (27·1)	33 (30·6)	21 (23)	0·237
Cardiovascular	100 (50·3)	46 (42·6)	54 (59)	0·019
Chronic renal disease	6 (3·0)	5 (4·6)	1 (1)	0·147
Cerebral/peripheral vascular disease	15 (7·5)	8 (7·4)	7 (8)	0·940
Liver failure/cirrhosis	1 (0·5)	1 (0·9)	0 (0)	1·000
Barrett's oesophagus	28 (14·1)	11 (10·2)	17 (19)	0·086
Diabetes	28 (14·1)	11 (10·2)	17 (19)	0·086
Chronic respiratory disease	37 (18·6)	18 (16·7)	19 (21)	0·447
Other significant co‐morbidity	44 (22·1)	24 (22·2)	20 (22)	0·967
**Cell type**				0·313
Invasive adenocarcinoma	189 (95·0)	101 (93·5)	88 (97)	
Adenosquamous carcinoma	8 (4·0)	6 (5·6)	2 (2)	
Squamous cell carcinoma	2 (1·0)	1 (0·9)	1 (1)	
**T category**				0·841
T1	1 (0·5)	0 (0)	1 (1)	
T2	62 (31·2)	35 (32·4)	27 (30)	
T3	107 (54·3)	57 (52·8)	50 (55)	
T4	28 (14·1)	15 (13·9)	13 (14)	
Missing	1 (0·5)	1 (0·1)	0 (0)	
**N category**				0·328
N0	73 (36·7)	43 (39·8)	30 (33)	
N1	78 (39·2)	40 (37·0)	38 (42)	
N2	40 (20·1)	21 (19·4)	19 (21)	
N3	7 (3·5)	3 (2·8)	4 (4)	
Missing	1 (0·5)	1 (0·9)	0 (0)	
**M category**				0·356
M0	197 (99·0)	106 (98·1)	91 (100)	
M1	1 (0·5)	1 (0·9)	0 (0)	
Missing	1 (0·5)	1 (0·9)	0 (0)	
**Tumour site**				0·814
Oesophagus	68 (34·2)	37 (34·3)	31 (34)	
Gastro‐oesophageal junction	106 (53·3)	56 (51·9)	50 (55)	
Stomach	25 (12·6)	15 (13·9)	10 (11)	
**Tumour subsite**				0·900
Lower third	68 (34·2)	37 (34·3)	31 (34)	
Siewert 1	54 (27·1)	29 (26·9)	25 (27)	
Siewert 2	37 (18·6)	19 (17·6)	18 (20)	
Siewert 3	16 (8·0)	9 (8·3)	7 (8)	
Body	14 (7·0)	5 (4·6)	9 (10)	
Antrum	4 (2·0)	4 (3·7)	0 (0)	
Pylorus	5 (2·5)	4 (3·7)	1 (1)	
Missing	1 (0·5)	1 (0·9)	0 (0)	
**Regimen**				0·107
CX	55 (27·6)	23 (21·3)	32 (35)	
ECX	94 (47·2)	56 (51·9)	38 (42)	
Other	50 (25·1)	29 (26·9)	21 (23)	
**Cycles of chemotherapy**				0·248
1	10 (5·0)	7 (6·5)	3 (3)	
2	66 (33·2)	29 (26·9)	37 (41)	
3	122 (61·3)	72 (66·7)	50 (55)	
4	1 (0·5)	0 (0·0)	1 (1)	
**Feeding tube during NAT**				
Jejunostomy	45 (22·6)	25 (23·1)	20 (22)	0·844§
Nasogastric tube	3 (1·5)	2 (1·9)	1 (1)	0·644§
**Procedure type**				0·320
Left thoracoabdominal approach	89 (44·7)	46 (42·6)	43 (47)	
2‐phase (Ivor Lewis)	55 (27·6)	30 (27·8)	25 (27)	
3‐phase (McKeown)	6 (3·0)	3 (2·8)	3 (3)	
Thoracotomy (open and shut)	3 (1·5)	3 (2·8)	0 (0)	
Total gastrectomy	11 (5·5)	5 (4·6)	6 (7)	
Extended total gastrectomy	11 (5·5)	4 (3·7)	7 (8)	
Distal gastrectomy	13 (6·5)	11 (10·2)	2 (2)	
Laparotomy (open and shut)	11 (5·5)	6 (5·6)	5 (5)	
**Approach**				0·978
Open operation	177 (88·9)	96 (88·9)	81 (89)	
Laparoscopic/thoracoscopic	22 (11·1)	12 (11·1)	10 (11)	
**Time of CT before event (days)†**				
Before start of NAT	35 (25–46)	36 (25–47)	34 (23–45)	0·254
Before surgery	30 (21–39)	30 (21–40)	32 (26–40)	0·115
Time between scans	105 (75–135)	107 (91–123)	104 (91–118)	0·194

Values in parentheses are percentages unless indicated otherwise; values are

* median (range) and †median (i.q.r.). SMI, skeletal muscle index; (E)CX, (epirubicin)–cisplatin–capecitabine; NAT, neoadjuvant therapy.

‡ Mann–Whitney *U* test, except §χ^2^ test.

**Table 2:** Body composition changes during neoadjuvant therapy

	**Before NAT (n = 199)**	**Before surgery (n = 199)**	**P** **‡**
Height (m)*	1·70(0·09)		
Weight (kg)	78·35 (67·33–89·38)		
BMI (kg/m^2^)	27·03 (23·78–30·28)		
SMI (cm^2^/m^2^)*	51·87(10·31)	49·19(9·71)	< 0·001§
SMA (cm^2^)*	150·41(33·61)	142·60(31·94)	< 0·001§
Sarcopenia†	84 (42·2)	108 (54·3)	< 0·001¶
SFI (cm^2^/m^2^)	67·11 (42·59–91·65)	59·10 (41·54–76·67)	< 0·001
SFA (cm^2^)	165·09 (113·2–216·97)	172·40 (123·65–221·15)	0·617
VFI (cm^2^/m^2^)	61·81 (37·30–86·32)	60·76 (37·55–83·97)	0·063
VFA (cm^2^)	177·15 (37·82–196·49)	167·48 (98·60–236·36)	0·060

Values are median (i.q.r.) unless indicated otherwise;

* values are mean(s.d.);

† values in parentheses are percentages. NAT, neoadjuvant therapy; SMI, skeletal muscle index; SMA, skeletal muscle area; SFI, subcutaneous fat index; SFA, subcutaneous fat area; VFI, visceral fat index; VFA, visceral fat area.

‡ Wilcoxon test, except

§ paired *t* test and

¶ McNemar test.

Patient groups with an SMI decrease of 5 per cent or less and more than 5 per cent are compared in *Table* *1*. Patients with a higher rate of muscle mass depletion were significantly older than those with a lower rate (median 67·6 *versus* 65·6 years respectively; *P* = 0·009) and were more likely to be men (89·0 *versus* 71·3 per cent respectively; *P* = 0·002). In addition, the patients with a higher rate of muscle depletion had a higher percentage of cardiovascular co‐morbidity (59·3 *versus* 42·6 per cent; *P* = 0·019). The median interval from staging CT to the start of neoadjuvant therapy was 35 (i.q.r. 25–46) days. The median interval from preoperative CT to surgery was 30 (i.q.r. 21–39) days. There were no significant differences between the groups in the timing of staging CT (*P* = 0·254) or preoperative CT (*P* = 0·115) (*Table* *1*). The median interval between staging and preoperative CT was 105 (i.q.r. 75–135) days, and a majority of patients (122, 61·3 per cent) received three cycles of chemotherapy (*Table* *1*).

### Body composition changes during neoadjuvant chemotherapy

**Fig. 1:**
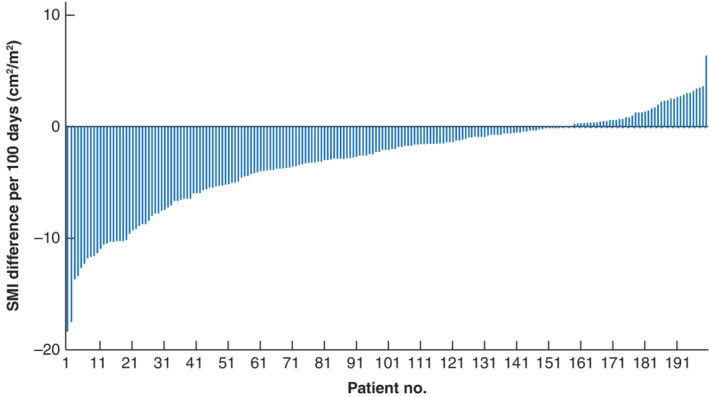
Waterfall plot of the difference per 100 days in skeletal muscle index during neoadjuvant therapy in 199 patients SMI, skeletal muscle index.

Baseline characteristics demonstrated a population with a median BMI of 25·75 and 27·41 kg/m^2^ for women and men respectively. Some 132 (66·3 per cent) of the 199 included patients were overweight (BMI 25 kg/m^2^ or above) (*Table* *2*). In total, 108 patients (54·3 per cent) had a SMI decrease of 5 per cent or less and 91 (45·7 per cent) lost more than 5 per cent (*Table* *1* and *Fig*. *1*). In men, the mean SMI decreased by 5·9 per cent (from 54·46 to 51·27 cm^2^/m^2^; *P* < 0·001) (*Table* *S1*, supporting information). In women, there was a 1·7 per cent decrease in SMI, which was not statistically significant (from 41·87 to 41·15 cm^2^/m^2^; *P* = 0·107). Before the start of NAT, 17 of the 41 women (41 per cent) and 67 of the 158 men (42·4 per cent) were sarcopenic. Before surgery this increased to 19 (46 per cent) and 89 (56·3 per cent) respectively. This increase in sarcopenia was significant only for men (*P* < 0·001). Changes in subcutaneous and visceral fat mass are shown in *Table* *S1* (supporting information).

### Postoperative complications

In total, 101 patients (50·8 per cent) had at least one postoperative complication (Clavien–Dindo grade I–V), and 27 (13·6 per cent) had a severe complication (grade IIIa or above) (*Table* *3*). Pneumonia was the most common postoperative complication, with 52 cases (26·1 per cent). Thirty‐ and 90‐day mortality occurred in only two (1·0 per cent) and three (1·5 per cent) patients respectively. When comparing patients with a change in SMI of more than 5 per cent or 5 per cent or less, no significant differences were detected between the two groups regarding total (49 *versus* 51·9 per cent respectively; *P* = 0·736) and severe (11 *versus* 15·7 per cent; *P* = 0·329) complications. A significant difference was found in the incidence of wound infection, with a higher rate in patients with an SMI change of 5 per cent or less (9·3 per cent *versus* 0 per cent in the group with a greater decrease in SMI; *P* = 0·003).

**Table 3:** Overview of postoperative complications and mortality

		Decrease in SMI (%)		
	Total (*n* = 199)	≤ 5 (*n* = 108)	> 5 (*n* = 91)	*P* *
**Complications**				
Total complications (CD I–V)	101 (50·8)	56 (51·9)	45 (49)	0·736
Severe complications (CD ≥ IIIa)	27 (13·6)	17 (15·7)	10 (11)	0·329
Anastomotic leak	8 (4·0)	7 (6·5)	1 (1)	0·054
Pneumonia	52 (26·1)	29 (26·9)	23 (25)	0·801
Pleural effusion	14 (7·0)	11 (10·2)	3 (3)	0·058
Atrial dysrhythmia	20 (10·1)	10 (9·3)	10 (11)	0·686
Deep venous thrombosis	3 (1·5)	3 (2·8)	0 (0)	0·252
Pulmonary embolism	5 (2·5)	2 (1·9)	3 (3)	0·662
Wound infection	10 (5·0)	10 (9·3)	0 (0)	0·003
Vocal cord nerve injury	5 (2·5)	3 (2·8)	2 (2)	0·795
Chyle leak	17 (8·5)	10 (9·3)	7 (8)	0·694
Reoperation	3 (1·5)	2 (1·9)	1 (1)	1·000
**Mortality**				
30‐day	2 (1·0)	1 (0·9)	1 (1)	1·000
90‐day	3 (1·5)	2 (1·9)	1 (1)	1·000

Values in parentheses are percentages. SMI, skeletal muscle index; CD, Clavien–Dindo grade.

* χ^2^ or Fisher's exact test.

### Analysis of changes in skeletal muscle index and total postoperative complications

Univariable analysis revealed that a decrease in the SMI greater than 5 per cent during NAT was not associated with a higher risk of postoperative complications (OR 0·91, 95 per cent c.i. 0·52 to 1·59; *P* = 0·736) (*Table* *4*). Neither did the 10 per cent of patients with the greatest decrease in SMI during NAT show a higher risk of total postoperative complications (OR 0·99, 0·39 to 2·46; *P* = 0·962). Sarcopenia before the start of NAT (OR 1·03, 0·59 to 1·81; *P* = 0·916) or surgery (OR 1·02, 0·58 to 1·77; *P* = 0·958) did not have significant prognostic value for total complications. Multiple regression analysis did not show a predictive value for an SMI decrease greater than 5 per cent (OR 0·81, 0·45 to 1·46; *P* = 0·484) (*Table* *4*). The complete results of the univariable and multiple regression analyses can be seen in *Table* *S2* (supporting information).

**Table 4:** Univariable and multiple regression analysis of changes in skeletal muscle index and postoperative complications

	Univariable analysis		Multiple regression analysis	
	Odds ratio	P	Odds ratio	P
**Total postoperative complications** *				
SMI change > 5%	0·91 (0·52, 1·59)	0·736	0·81 (0·45, 1·46)	0·484
10% of patients with greatest SMI decrease	0·99 (0·39, 2·46)	0·962		
Sarcopenia				
Before NAT	1·03 (0·59, 1·81)	0·916		
Before surgery	1·02 (0·58, 1·77)	0·958		
**Severe postoperative complications** **†**				
SMI change >5%	0·66 (0·29, 1·53)	0·329	0·58 (0·24, 1·39)	0·222
10% of patients with greatest SMI decrease	0·31 (0·04, 2·43)	0·241		
Sarcopenia				
Before NAT	0·93 (0·41, 2·13)	0·868		
Before surgery	0·89 (0·40, 2·01)	0·786		

Values in parentheses are 95 per cent confidence intervals.

* Clavien–Dindo grade I–V;

† Clavien–Dindo grade IIIa and above. SMI, skeletal muscle index; NAT, neoadjuvant therapy.

### Analysis of changes in skeletal muscle index and severe postoperative complications

Patients with a SMI decrease greater than 5 per cent during NAT did not have an increased risk of developing severe postoperative complications (Clavien–Dindo grade IIIa and above) (OR 0·66, 95 per cent c.i. 0·29 to 1·53; *P* = 0·329) (*Table* *4*). Similarly, the 10 per cent of the patients with the greatest SMI decrease during NAT did not show a significantly higher risk of severe complications. Neither sarcopenia before the start of NAT (OR 0·93, 0·41 to 2·13; *P* = 0·868), nor sarcopenia before surgery (OR 0·89, 0·40 to 2·01; *P* = 0·786) had a significant prognostic value. Accounting for other clinical variables, multiple regression analysis also failed to demonstrate that SMI changes predicted postoperative outcomes (*Table* *S2*, supporting information).

### Analysis of changes in skeletal muscle index and three specific postoperative complications

Univariable and multiple regression analyses were performed for three specific postoperative complications: pneumonia, anastomotic leak and atrial fibrillation (*Tables* *S3* *–* *S5*, supporting information). A decrease of more than 5 per cent in SMI during NAT was not predictive for any of these complications (pneumonia: OR 0·78, 95 per cent c.i. 0·40 to 1·51, *P* = 0·455; anastomotic leak: OR 0·14, 0·02 to 1·23, *P* = 0·076; atrial fibrillation: OR 1·03, 0·39 to 2·75, *P* = 0·953).

## Discussion

The use of NAT was associated with a significant decrease in SMI. Patients with the greatest reduction in SMI were not at increased risk of postoperative complications. Sarcopenia either before NAT or surgery did not predict postoperative complications. No association was found for specific complications including pneumonia, anastomotic leak and atrial fibrillation.

The decrease in SMI in the present study was comparable to that found in previous studies^5^, ^16^, ^17^. Others have demonstrated a significant predictive value of muscle mass depletion during NAT for overall survival. One study^17^ showed a significant predictive value of skeletal muscle area loss (above 6 per cent) in palliative foregut cancer for overall survival, whereas another^16^ demonstrated that loss of skeletal muscle mass during NAT had prognostic value for overall survival, although without significant differences in postoperative complications between the groups with the highest and lowest degree of muscle mass loss. Although muscle mass loss during NAT seems to have a negative impact on overall survival, it does not appear to predict postoperative complications.

Dysphagia and weight loss are the most common symptoms of gastro‐oesophageal cancer^16^. This is reflected in the relatively high prevalence of sarcopenia in the present study population (54·3 per cent) compared with other abdominal malignancies^18^. Multiple factors may be responsible for muscle mass depletion during NAT: immobility, feeding problems, loss of appetite, and effects from cancer‐related cytokines^19^. In gastro‐oesophageal cancer it is possible that sarcopenia is predominantly an effect of dysphagia and inadequate nutrition, whereas in other cancers it might be related predominantly to cancer burden or paraneoplastic effects. Different mechanisms for the development of sarcopenia might influence specific outcomes, such as surgical complication rates.

Loss of skeletal muscle is not the only indicator of functional depletion^20^. Physical tests such as handgrip strength or walking speed are combined with muscle mass to diagnose sarcopenia according to the current consensus of the European Working Group on Sarcopenia and Older People^21^. In the present study, no data on physical performance during NAT were available. The combined measurement of muscle mass and muscle function may be a better predictor of short‐term surgical outcomes.

Both complications after surgery and postoperative loss of skeletal muscle mass are associated with impaired survival in oesophageal cancer^22^. Although the present study showed that a significant decrease in skeletal muscle mass occurred during NAT in patients with gastro‐oesophageal cancer before surgery, this change was not associated with an increased risk of postoperative complications. Sarcopenia related to the effects of the primary tumour and NAT is not a likely explanation for the effect of postoperative complications on long‐term survival.

## Supporting information


**Table S1** Body composition changes during neoadjuvant therapy in 41 women and 158 men
**Table S2** Univariable and multiple regression analysis of changes in skeletal muscle index and total (Clavien–Dindo grade I–V) and severe (Clavien–Dindo grade IIIa and above) postoperative complications
**Table S3** Univariable and multiple regression analysis of changes in skeletal muscle index and pneumonia
**Table S4** Univariable and multiple regression analysis of changes in skeletal muscle index and anastomotic leak
**Table S5** Univariable and multiple regression analysis of changes in skeletal muscle index and atrial fibrillation

## References

[1] Bray F , Ferlay J , Soerjomataram I , Siegel RL , Torre LA , Jemal A . Global cancer statistics 2018: GLOBOCAN estimates of incidence and mortality worldwide for 36 cancers in 185 countries. CA Cancer J Clin 2018; 68: 394–424. 30207593 10.3322/caac.21492

[2] Pressoir M , Desné S , Berchery D , Rossignol G , Poiree B , Meslier M *et al* Prevalence, risk factors and clinical implications of malnutrition in French Comprehensive Cancer Centres. Br J Cancer 2010; 102: 966–971. 20160725 10.1038/sj.bjc.6605578 PMC2844030

[3] Thrumurthy SG , Chaudry MA , Hochhauser D , Mughal M. The diagnosis and management of gastric cancer. BMJ 2013; 347: f6367. 24191271 10.1136/bmj.f6367

[4] Gil KM , Frasure HE , Hopkins MP , Jenison EL , von Gruenigen VE . Body weight and composition changes in ovarian cancer patients during adjuvant chemotherapy. Gynaecol Oncol 2006; 103: 247–252. 10.1016/j.ygyno.2006.03.005 16690107

[5] Awad S , Tan BH , Cui H , Bhalla A , Fearon KCH , Parsons SL *et al* Marked changes in body composition following neoadjuvant chemotherapy for oesophagogastric cancer. Clin Nutr 2012; 31: 74–77. 21875767 10.1016/j.clnu.2011.08.008

[6] Cruz‐Jentoft AJ , Sayer AA . Sarcopenia. Lancet 2019; 393: 2636–2646. 31171417 10.1016/S0140-6736(19)31138-9

[7] Kamarajah SK , Bundred J , Tan BHL . Body composition assessment and sarcopenia in patients with gastric cancer: a systematic review and meta‐analysis. Gastric Cancer 2018; 22: 10–22. 30276574 10.1007/s10120-018-0882-2

[8] Boshier PR , Heneghan R , Markar SR , Baracos VE , Low DE . Assessment of body composition and sarcopenia in patients with esophageal cancer: a systematic review and meta‐analysis. Dis Esophagus 2018; 31: 1–11. 10.1093/dote/doy047 29846548

[9] Blauwhoff‐Buskermolen S , Versteeg KS , van der Schueren MAED , den Braver NR , Berkhof J , Langius JAE *et al* Loss of muscle mass during chemotherapy is predictive for poor survival of patients with metastatic colorectal cancer. J Clin Oncol 2019; 34: 1339–1344. 10.1200/JCO.2015.63.6043 26903572

[10] Rutten IJG , van Dijk DPJ , Kruitwagen RFPM , Beets‐tan RGH , Olde SWM , Van Gorp T . Loss of skeletal muscle during neoadjuvant chemotherapy is related to decreased survival in ovarian cancer patients. J Cachexia Sarcopenia Muscle 2016; 7: 458–466. 27030813 10.1002/jcsm.12107 PMC4782251

[11] Clavien PA , Barkun J , de Oliveira ML , Vauthey JN , Dindo D , Schulick RD *et al* The Clavien–Dindo classification of surgical complications: five‐year experience. Ann Surg 2009; 250: 187–196. 19638912 10.1097/SLA.0b013e3181b13ca2

[12] Low DE , Kuppusamy MK , Alderson D , Cecconello I , Chang AC , Darling G *et al* Benchmarking complications associated with esophagectomy. Ann Surg 2019; 269: 291–298. 29206677 10.1097/SLA.0000000000002611

[13] Mullie L , Afilalo J . CoreSlicer: a web toolkit for analytic morphomics. BMC Med Imaging 2019; 19: 15. 30744586 10.1186/s12880-019-0316-6 PMC6371488

[14] Portal D , Hofstetter L , Eshed I , Dan‐Lantsman C , Sella T , Urban D *et al* L3 skeletal muscle index (L3SMI) is a surrogate marker of sarcopenia and frailty in non‐small cell lung cancer patients. Cancer Manag Res 2019; 11: 2579–2588. 31114324 10.2147/CMAR.S195869 PMC6497853

[15] Prado CMM , Lieffers JR , McCargar LJ , Reiman T , Sawyer MB , Martin L *et al* Prevalence and clinical implications of sarcopenic obesity in patients with solid tumours of the respiratory and gastrointestinal tracts: a population‐based study. Lancet Oncol 2008; 9: 629–635. 18539529 10.1016/S1470-2045(08)70153-0

[16] Järvinen T , Ilonen I , Kauppi J , Salo J , Räsänen J . Loss of skeletal muscle mass during neoadjuvant treatments correlates with worse prognosis in esophageal cancer: a retrospective cohort study. World J Surg Oncol 2018; 16: 27. 29433514 10.1186/s12957-018-1327-4 PMC5809976

[17] Daly LE , Ní Bhuachalla ÉB , Power DG , Cushen SJ , James K , Ryan AM . Loss of skeletal muscle during systemic chemotherapy is prognostic of poor survival in patients with foregut cancer. J Cachexia Sarcopenia Muscle 2018; 9: 315–325. 29318756 10.1002/jcsm.12267 PMC5879982

[18] Morishita S . Prevalence of sarcopenia in cancer patients: review and future directions. Int J Phys Med Rehabil 2016; 04: 1–8.

[19] Ryan AM , Power DG , Daly L , Cushen SJ , Bhuachalla ĒN , Prado CM . Cancer‐associated malnutrition, cachexia and sarcopenia: the skeleton in the hospital closet 40 years later. Proc Nutr Soc 2019; 75: 199–211. 10.1017/S002966511500419X 26786393

[20] Cesari M , Araujo de Carvalho I , Amuthavalli Thiyagarajan J , Cooper C , Martin FC , Reginster J‐Y *et al* Evidence for the domains supporting the construct of intrinsic capacity. J Gerontol A Biol Sci Med Sci 2018; 73: 1653–1660. 29408961 10.1093/gerona/gly011

[21] Cruz‐Jentoft A , Bahat G , Bauer J , Boirie Y , Bruyère O , Tommy C *et al*; Writing Group for the European Working Group on Sarcopenia in Older People 2 (EWGSOP2), and the Extended Group for EWGSOP2. Sarcopenia: revised European consensus on definition and diagnosis. Age Ageing 2019; 48: 16–31.30312372

[22] Mayanagi S , Tsubosa Y , Omae K , Niihara M , Uchida T , Tsushima T *et al* Negative impact of skeletal muscle wasting after neoadjuvant chemotherapy followed by surgery on survival for patients with thoracic esophageal cancer. Ann Surg Oncol 2017; 24: 3741–3747. 28861809 10.1245/s10434-017-6020-2 PMC5658455

