# Supplementary material for: Impact on postoperative complications of changes in skeletal muscle mass during neoadjuvant chemotherapy for gastro‐oesophageal cancer
Source: BJS Open. 2020 Aug 25;4(5):847–54. doi: 10.1002/bjs5.50331 (PMC7528528; doi:10.1002/bjs5.50331)
Supplement: Supplementary file 1 — Table S1 Body composition changes during neoadjuvant therapy in 41 women and 158 men Table S2 Univariable and multiple regression analysis of changes in skeletal muscle index and total (Clavien–Dindo grade I–V) and severe (Clavien–Dindo grade IIIa and above) postoperative complications Table S3 Univariable and multiple regression analysis of changes in skeletal muscle index and pneumonia Table S4 Univariable and multiple regression analysis of changes in skeletal muscle index and anastomotic leak Table S5 Univariable and multiple regression analysis of changes in skeletal muscle index and atrial fibrillation [file BJS5-4-847-s001.docx]

**BJS5_50331**

**Impact on postoperative complications of changes in skeletal muscle mass during neoadjuvant chemotherapy for gastro-oesophageal cancer**

**R. B. den Boer, K. I. Jones, S. Ash, G. I. van Boxel, R. S. Gillies, T. O’Donnell, J. P. Ruurda, B. Sgromo, M. A. Silva and N. D. Maynard**

**Table S1** Body composition changes during neoadjuvant therapy in 41 women and 158 men

| Variable | Pre NAT (female) | Pre surgery (female) | P value | Pre NAT (male) | Pre surgery (male) | P value |
| --- | --- | --- | --- | --- | --- | --- |
| Height in m (mean, SD) | 1.60 (0.08) |  |  | 1.73 (0.07) |  |  |
| Weight in kg (median, IQR) | 64.00 (16.82) |  |  | 80.83 (19.96 |  |  |
| BMI in kg/m^2^ (median, IQR) | 25.75 (7.00) |  |  | 27.41 (6.04) |  |  |
| SMI in cm^2^/m^2^ (mean, SD) | 41.87 (1.21) | 41.15 (1.19) | 0.107 | 54.46 (9.26) | 51.27 (9.10) | **0.000** |
| SMA in cm^2^ (mean, SD) | 106.92 (19.00) | 105.18 (19.80) | 0.147 | 161.69 (26.67) | 152.31 (26.95) | **0.000** |
| Sarcopenia (n, %) | 17 (41.5%) | 19 (46.3%) | 0.625 | 67 (42.4%) | 89 (56.3%) | **0.000** |
| SFI in cm^2^/m^2^ (median, IQR) | 95,04 (97,78) | 75,52 (71,02) | **0.005** | 63,20 (38,19) | 56,40 (30.63) | **0.000** |
| SFA in cm^2^ (median, IQR) | 190.25 (204.53) | 183.78 (156.73) | 0.206 | 159.43 (92.34) | 168.97 (89.14) | 0.905 |
| VFI in cm^2^ (median, IQR) | 43.31 (50.84) | 45.00 (37.77) | 0.184 | 66,17 (47.23) | 64,78 (45.15) | **0.014** |
| VFA in cm^2^ (median, IQR) | 105.04 (113.81) | 107.73 (83.40) | 0.197 | 196,10 (154.81) | 194,55 (96.67) | **0.016** |

**IQR, Interquartile range; SD, standard deviation; SMA, skeletal muscle area; SMI, skeletal muscle index; SFA, subcutaneous fat area; SFI, subcutaneous fat index; VFA, visceral fat area; VFI, visceral fat index.**

**Table S2** Univariable and multiple regression analysis of changes in skeletal muscle index and total (Clavien–Dindo grade I–V) and severe (Clavien–Dindo grade IIIa and above) postoperative complications

Total complications Univariable analysis Multiple regression analysis

| Variable | Odds ratio | Confidence interval 95% | p value | Odds ratio | Confidence interval 95% | p value |
| --- | --- | --- | --- | --- | --- | --- |
| Male gender | 1.395 | 0.699 - 2.785 | 0.343 | 1.476 | 0.718 – 3.033 | 0.289 |
| No comorbidity | 0.879 | 0.471 - 1.642 | 0.686 |  |  |  |
| Cardiovascular comorbidity | 1.222 | 0.701 - 2.128 | 0.479 |  |  |  |
| Cerebral/peripheral vascular comorbidity | 0.33 | 0.101 - 1.074 | 0.055 |  |  |  |
| Diabetes | 0.650 | 0.293 - 1.444 | 0.288 |  |  |  |
| Chronic respiratory comorbidity | 1.559 | 0.756 - 3.219 | 0.227 |  |  |  |
| 2 or more comorbidities | 1.049 | 0.590 - 1.865 | 0.870 | 0.962 | 0.531 – 1.742 | 0.897 |
| Laparoscopic vs. open approach | 0.978 | 0.403 - 2.371 | 0.960 |  |  |  |
| SMI change >5% | 0.908 | 0.520 - 1.587 | 0.736 | 0.811 | 0.451 – 1.459 | 0.484 |
| Greatest 10% SMI decrease | 0.987 | 0.388 - 2.464 | 0.962 |  |  |  |
| Sarcopenia pre NAT | 1.031 | 0.587 - 1.809 | 0.916 |  |  |  |
| Sarcopenia pre surgery | 1.015 | 0.581 - 1.773 | 0.958 |  |  |  |
| Older than 65 year | 1.219 | 0.698 - 2.130 | 0.486 | 1.129 | 0.627 – 2.033 | 0.686 |
| BMI >25 kg/m^2^ | 0.795 | 0.439 - 1.442 | 0.451 |  |  |  |
| ASA III | 1.776 | 0.948 - 3.329 | 0.072 | 1.81 | 0.943 – 3.472 | 0.074 |
| Positive lymph node | 1.004 | 0.564 - 1.788 | 0.988 |  |  |  |
| Cancer stage >II | 1.019 | 0.584 - 1.776 | 0.948 | 1.118 | 0.630 – 1.984 | 0.704 |

Severe complications Univariate analysis Multiple regression analysis

| Variable | Odds ratio | Confidence interval 95% | p value | Odds ratio | Confidence interval 95% | p value |
| --- | --- | --- | --- | --- | --- | --- |
| Male gender | 2.252 | 0.643 - 7.884 | 0.194 | 2.803 | 0.779 – 10.085 | 0.115 |
| No comorbidity | 0.744 | 0.283 - 1.956 | 0.548 |  |  |  |
| Cardiovascular comorbidity | 1.089 | 0.484 - 2.453 | 0.836 |  |  |  |
| Cerebral/peripheral vascular comorbidity | 0.985 | 0.210 - 4.626 | 0.984 |  |  |  |
| Diabetes | 0.433 | 0.097 - 1.934 | 0.260 |  |  |  |
| Chronic respiratory comorbidity | 1.001 | 0.353 - 2.845 | 0.998 |  |  |  |
| 2 or more comorbidities | 1.215 | 0.515 - 2.864 | 0.656 | 1.437 | 0.591 – 3.495 | 0.424 |
| Laparoscopic vs. open approach | 1.013 | 0.279 - 3.686 | 0.984 |  |  |  |
| SMI change >5% | 0.661 | 0.286 - 1.525 | 0.329 | 0.581 | 0.243 – 1.389 | 0.222 |
| Greatest 10% SMI decrease | 0.312 | 0.040 - 2.430 | 0.241 |  |  |  |
| Sarcopenia pre NAT | 0.932 | 0.409 - 2.128 | 0.868 |  |  |  |
| Sarcopenia pre surgery | 0.894 | 0.397 - 2.014 | 0.786 |  |  |  |
| Older than 65 year | 0.612 | 0.271 - 1.385 | 0.236 | 0.619 | 0.263 – 1.458 | 1.458 |
| BMI >25 kg/m^2^ | 0.961 | 0.403 - 2.291 | 0.929 |  |  |  |
| ASA III | 0.544 | 0.195 - 1.515 | 0.238 | 0.559 | 0.194 – 1.609 | 0.281 |
| Positive lymph node | 1.185 | 0.503 - 2.795 | 0.698 |  |  |  |
| Cancer stage >II | 0.866 | 0.384 - 1.951 | 0.728 | 0.816 | 0.352 – 1.893 | 0.636 |

**Table S3** Univariable and multiple regression analysis of changes in skeletal muscle index and pneumonia

Univariable analysis Multiple regression analysis

| Variable | Odds ratio | Confidence interval 95% | p value | Odds ratio | Confidence interval 95% | p value |
| --- | --- | --- | --- | --- | --- | --- |
| Male gender | 1.934 | 0.799 - 4.681 | 0.138 | 2.029 | 0.813 – 5.061 | 0.129 |
| No comorbidity | 0.649 | 0.305 - 1.380 | 0.259 |  |  |  |
| Cardiovascular comorbidity | 1.215 | 0.645 - 2.291 | 0.546 |  |  |  |
| Cerebral/peripheral vascular comorbidity | 0.412 | 0.090 - 1.892 | 0.241 |  |  |  |
| Diabetes | 0.427 | 0.141 - 1.296 | 0.124 |  |  |  |
| Chronic respiratory comorbidity | 1.464 | 0.674 - 3.179 | 0.334 |  |  |  |
| 2 or more comorbidities | 1.304 | 0.669 - 2.542 | 0.435 | 1.243 | 0.623 – 2.479 | 0.537 |
| Laparoscopic vs. open approach | 0.814 | 0.284 - 2.328 | 0.700 |  |  |  |
| SMI change >5% | 0.921 | 0.488 - 1.741 | 0.801 | 0.775 | 0.397 – 1.513 | 0.455 |
| Greatest 10% SMI decrease | 2.248 | 0.850 - 5.942 | 0.096 |  |  |  |
| Sarcopenia pre NAT | 1.005 | 0.530 - 1.907 | 0.987 |  |  |  |
| Sarcopenia pre surgery | 0.977 | 0.518 - 1.843 | 0.943 |  |  |  |
| Older than 65 year | 1.455 | 0.763 - 2.774 | 0.254 | 1.252 | 0.636 – 2.463 | 0.516 |
| BMI >25 kg/m^2^ | 1.094 | 0.559 - 2.141 | 0.794 |  |  |  |
| ASA III | 1.574 | 0.796 - 3.113 | 0.191 | 1.413 | 0.698 – 2.863 | 0.337 |
| Positive lymph node | 0.732 | 0.383 - 1.398 | 0.344 |  |  |  |
| Gastrectomy vs. oesophagectomy | 0.402 | 0.159 - 1.019 | 0.049 |  |  |  |
| Cancer stage >II | 0.691 | 0.366 - 1.307 | 0.255 | 0.737 | 0.384 – 1.414 | 0.359 |

**Table S4** Univariable and multiple regression analysis of changes in skeletal muscle index and anastomotic leak

Univariable analysis Multiple regression analysis

| Variable | Odds ratio | Confidence interval 95% | P value | Odds ratio | Confidence interval 95% | P value |
| --- | --- | --- | --- | --- | --- | --- |
| Male gender | 1.854 | 0.222 - 15.512 | 0.563 | 2.835 | 0.329 – 24.417 | 0.343 |
| No comorbidity | 0.891 | 0.174 - 4.555 | 0.890 |  |  |  |
| Cardiovascular comorbidity | 0.316 | 0.062 - 1.607 | 0.145 |  |  |  |
| Cerebral/peripheral vascular comorbidity | 0.957 | 0.928 - 0.986 | 0.410 |  |  |  |
| Diabetes | 2.115 | 0.405 - 11.047 | 0.364 |  |  |  |
| Chronic respiratory comorbidity | 1.486 | 0.288 - 7.673 | 0.634 |  |  |  |
| 2 or more comorbidities | 0.986 | 0.229 - 4.251 | 0.985 | 1.257 | 0.278 – 5.679 | 0.766 |
| Laparoscopic vs. open approach | 2.850 | 0.529 - 15.079 | 0.199 |  |  |  |
| SMI change >5% | 0.160 | 0.019 - 1.328 | 0.054 | 0.144 | 0.017 – 1.225 | 0.076 |
| Greatest 10% SMI decrease | 0.926 | 0.926 - 0.986 | 0.348 |  |  |  |
| Sarcopenia pre NAT | 1.388 | 0.337 - 5.714 | 0.724 |  |  |  |
| Sarcopenia pre surgery | 0.837 | 0.203 - 3.443 | 1.000 |  |  |  |
| Older than 65 year | 0.481 | 0.112 - 2.071 | 0.316 | 0.510 | 0.112 – 2.334 | 0.386 |
| BMI >25 kg/m^2^ | 0.504 | 0.122 - 2.081 | 0.449 |  |  |  |
| ASA III | 0.868 | 0.170 - 4.436 | 0.865 | 1.001 | 0.182 – 5.512 | 0.999 |
| Positive lymph node | 0.972 | 0.225 - 4.192 | 0.970 |  |  |  |
| Gastrectomy vs. oesophagectomy | 0.523 | 0.063 - 4.370 | 0.543 |  |  |  |
| Cancer stage >II | 0.959 | 0.233 - 3.946 | 0.953 | 0.987 | 0.231 – 4.214 | 0.987 |

**Table S5** Univariable and multiple regression analysis of changes in skeletal muscle index and atrial fibrillation

Univariable analysis Multiple regression analysis

| Variable | Odds ratio | Confidence interval 95% | p value | Odds ratio | Confidence interval 95% | p value |
| --- | --- | --- | --- | --- | --- | --- |
| Male gender | 1.042 | 0.329 - 3.304 | 0.944 | 0.950 | 0.278 – 3.244 | 0.934 |
| No comorbidity | 0.645 | 0.206 - 0.874 | 0.449 |  |  |  |
| Cardiovascular comorbidity | 2.523 | 0.928 - 6.861 | 0.063 |  |  |  |
| Diabetes | 1.615 | 0.498 - 5.239 | 0.421 |  |  |  |
| Chronic respiratory comorbidity | 2.046 | 0.729 - 5.741 | 0.167 |  |  |  |
| 2 or more comorbidities | 2.569 | 0.825 - 8.000 | 0.094 | 2.341 | 0.730 – 7.509 | 0.152 |
| Laparoscopic vs. open approach | 0.883 | 0.191 - 4.092 | 0.874 |  |  |  |
| SMI change >5% | 1.210 | 0.480 - 3.050 | 0.686 | 1.030 | 0.385 – 2.752 | 0.953 |
| Greatest 10% SMI decrease | 1.059 | 0.226 - 4.959 | 0.942 |  |  |  |
| Sarcopenia pre NAT | 0.904 | 0.352 - 2.319 | 0.833 |  |  |  |
| Sarcopenia pre surgery | 1.297 | 0.506 - 3.325 | 0.588 |  |  |  |
| Older than 65 year | 3.669 | 1.190 - 11.499 | 0.017 | 3.618 | 1.124 – 11.649 | **0.019** |
| BMI >25 kg/m^2^ | 2.226 | 0.714 - 6.943 | 0.159 |  |  |  |
| ASA III | 1.137 | 0.414 - 3.126 | 0.803 | 0.806 | 0.280 - 2.320 | 0.690 |
| Positive lymph node | 1.408 | 0.516 - 3.841 | 0.502 |  |  |  |
| Gastrectomy vs. oesophagectomy | 0.386 | 0.086 - 1.735 | 0.199 |  |  |  |
| Cancer stage >II | 0.956 | 0.379 – 2.410 | 0.924 | 1.105 | 0.426 – 2.868 | 0.837 |
